# Foodborne Illness-Related Diagnostic Coding and Healthcare-Utilization Patterns in South Korea, 2023: A Nationwide Claims-Based Descriptive Study

**DOI:** 10.3390/healthcare14162466

**Published:** 2026-08-10

**Authors:** Seeun Choi, Junjoeng Hwang, Min Sik Choi, Su-Jin Kang, Kiyon Rhew

**Affiliations:** College of Pharmacy, Dongduk Women’s University, Seoul 02748, Republic of Korea; 20244093@dongduk.ac.kr (S.C.); 20211787@dongduk.ac.kr (J.H.); mschoi@dongduk.ac.kr (M.S.C.)

**Keywords:** diagnostic coding, nationwide study, administrative claims data, foodborne illness, healthcare utilization, HIRA

## Abstract

**Highlights:**

**What are the main findings?**

- A total of 6.83 million unique claims-defined episodes generated 7.75 million diagnostic category records; 79.3% of the records were unspecified.
- Viral and unspecified records were more frequently observed in younger age groups, whereas bacterial records were concentrated among adults aged 19–64 years.

**What are the implications of the main findings?**

- Nationwide claims data provide a complementary view of medically attended foodborne illness-related diagnoses recorded in routine healthcare practice.
- Claims-based patterns should not be interpreted as confirmed incidence, etiologic distribution, clinical severity, or regional and individual risk.

**Abstract:**

**Background**: Foodborne illness surveillance commonly focuses on reported outbreaks and laboratory-confirmed infections. Nationwide information on foodborne illness-related diagnoses recorded during routine healthcare encounters remains limited in South Korea. This study described the volume and distribution of claims-defined foodborne illness-related episodes and associated diagnostic category records documented in the 2023 HIRA database. **Methods**: This nationwide retrospective descriptive study used administrative reimbursement claims data from the Health Insurance Review and Assessment Service in 2023. Repeated qualifying encounters for the same patient were grouped into episodes using a 30-day gap rule. For the monthly analysis, each episode was counted once and assigned to the diagnostic category first recorded during the episode; other category-specific analyses used non-mutually exclusive diagnostic category records. Unique episodes were summarized by calendar month, whereas diagnostic category records were summarized by demographic characteristics, selected underlying diseases, healthcare institution type, and region. **Results**: A total of 6,829,577 unique claims-defined episodes generated 7,754,215 diagnostic category records because an episode could contribute to more than one diagnostic category. Unspecified records accounted for 79.3%, followed by bacterial records (12.1%), viral records (8.6%), and protozoal/other-cause records (<0.1%). Viral and unspecified records were more frequently observed in younger age groups, whereas bacterial records were concentrated among adults aged 19–64 years. Most records were associated with clinic visits (80.3%). Monthly episode counts showed variation, with the highest count observed in July and relatively high counts also observed in January, August, and September. Crude record rates based on healthcare institution location varied across regions. **Conclusions**: Foodborne illness-related diagnostic records were predominantly unspecified and were most frequently documented in clinics, suggesting limited etiologic specificity in routine diagnostic coding. Claims-based findings may complement existing surveillance but should not be interpreted as confirmed disease incidence or etiologic distribution.

## 1. Introduction

Foodborne illness remains an important public health concern because of its high frequency, broad clinical spectrum, and associated healthcare and societal costs [1,2,3,4]. Although many illnesses are self-limited, some patients require medical care, and foodborne infections may result in serious acute or long-term consequences, particularly among young children, older adults, and individuals with underlying medical conditions [2,3,4]. Characterizing foodborne illness at the population level is therefore important for understanding how these conditions are identified and managed within healthcare systems.

Surveillance of foodborne illness commonly relies on reported outbreaks, laboratory-confirmed infections, and public health investigations [5,6,7]. These systems are essential for detecting clusters, identifying causative pathogens, and implementing outbreak-control measures. However, outbreak-based surveillance may not fully represent sporadic or individually managed episodes presenting in routine healthcare settings. Patients with gastrointestinal symptoms may seek care without being epidemiologically linked to a recognized outbreak, and microbiological testing may not be performed in every clinical encounter.

Healthcare claims data provide a complementary source of information on medically attended episodes across a broad range of patients and healthcare institutions. Because claims databases contain diagnostic and healthcare utilization information collected in routine practice, they can be used to describe how foodborne illness-related conditions are recorded and managed at the population level [8,9]. Nevertheless, administrative claims are generated primarily for reimbursement and clinical documentation rather than for epidemiologic confirmation [10,11,12]. Diagnostic codes may be influenced by coding practices, reimbursement requirements, and the availability of diagnostic testing. In addition, claims data generally do not include microbiological test results, detailed food-exposure histories, confirmed transmission routes, or validated clinical outcomes [8,9,10]. Accordingly, the claims-defined episodes in this study represent administrative records containing selected diagnostic codes rather than epidemiologically confirmed cases and should not be interpreted as estimates of the true incidence or actual burden of foodborne illness.

In South Korea, surveillance of gastrointestinal, waterborne, and foodborne infectious diseases includes mandatory and sentinel reporting as well as outbreak investigations [7,11,12,13]. Although this approach is critical for outbreak detection and public health response, nationwide information on foodborne illness-related diagnostic episodes recorded during routine healthcare encounters remains limited. A descriptive analysis of claims-defined episodes may provide additional information on the distribution of recorded diagnoses across age groups, healthcare settings, calendar months, and regions. Such findings should be interpreted as patterns of healthcare utilization and diagnostic coding rather than as estimates of pathogen-specific disease occurrence or community-level incidence.

The primary research question was: How many claims-defined foodborne illness-related episodes and diagnostic category records were documented in the 2023 HIRA database, and how were they distributed across diagnostic categories, demographic groups, healthcare institution types, calendar months, and healthcare-institution locations? Accordingly, this study aimed to describe administrative diagnostic coding and healthcare-utilization patterns for foodborne illness-related conditions documented in the 2023 HIRA database. This study was not intended to estimate laboratory-confirmed incidence, the true epidemiological or healthcare system burden of foodborne illness, identify causal determinants, or establish recurring seasonal patterns.

## 2. Materials and Methods

### 2.1. Study Design and Data Source

This nationwide retrospective descriptive study used customized administrative reimbursement claims data provided by the Health Insurance Review and Assessment Service (HIRA) in South Korea. Claims-defined foodborne illness-related episodes were identified between 1 January and 31 December 2023. Claims from the preceding 2 years were additionally used to ascertain selected underlying diseases.

South Korea operates a mandatory national health insurance system, and HIRA collects reimbursement claims covering nearly the entire Korean population. The customized research dataset contained de-identified information on patient demographics, insurance type, diagnoses, healthcare utilization, and prescription records. It did not include microbiological confirmation, detailed food-exposure information, outbreak attribution, or validated clinical outcomes.

Claims-defined episodes, rather than individual patients, were used to construct the study data. An individual patient could therefore contribute more than one episode during the study period. All data were de-identified before analysis.

This study was approved by the Institutional Review Board of Dongduk Women’s University (IRB No. DDWU2505-04; approval date: 30 April 2025). The requirement for informed consent was waived because the study used de-identified retrospective claims data.

### 2.2. Episode Definition, Diagnostic Classification, and Study Variables

Eligible diagnostic codes were defined according to the eighth revision of the Korean Standard Classification of Diseases and Causes of Death (KCD-8). The 30-day interval was used as an operational claims-based window to group closely spaced healthcare encounters that could represent continued or follow-up care for the same illness episode; a similar definition has been used in an electronic health record study of medically attended acute gastroenteritis [14]. For encounters accompanied by a prescription, the end of the medication supply period was used as the reference date. A subsequent qualifying encounter occurring less than 30 days after the reference date was considered part of the same episode, whereas an interval of 30 days or longer defined a new episode. For encounters without a prescription, the healthcare encounter date was used as the reference date. The index date of an episode was defined as the date on which the episode began.

Both primary and secondary diagnosis positions were used to identify eligible episodes to capture healthcare encounters in which a qualifying diagnostic code was documented during routine care. This approach was intended to provide a broad descriptive assessment of claims-defined records rather than to restrict the analysis to diagnoses recorded as the principal reason for the encounter. Accordingly, the identified episodes represented healthcare encounters associated with relevant diagnostic codes and were not necessarily laboratory-confirmed or epidemiologically confirmed foodborne illnesses.

Claims-defined episodes were classified into bacterial, viral, protozoal/other-cause, and unspecified diagnostic categories according to the KCD-8 codes presented in Supplementary Table S1. The diagnostic categories were not mutually exclusive. When an episode contained qualifying codes from more than one diagnostic category, it contributed once to each applicable category.

Selected underlying diseases included malignant neoplasms (C00–C97), diabetes mellitus (E10–E14), HIV/AIDS (B20–B24), and transplanted organs and tissues (Z94). Underlying diseases were identified from healthcare claims recorded during the 2-year look-back period preceding the index date of each episode. A patient with at least one claim containing a relevant diagnostic code during this period was classified as having the corresponding underlying disease.

The descriptive variables included sex, age group, insurance type, selected underlying disease, healthcare institution type, calendar month, and region. Age was categorized as <2, 2–5, 6–11, 12–18, 19–64, and ≥65 years. Insurance type was classified as National Health Insurance or Medical Aid. Healthcare institutions were categorized as clinics, hospitals, general hospitals, tertiary hospitals, or other institutions. Calendar month was assigned according to the index date of the episode. Region was defined according to the location of the healthcare institution at which the episode was recorded rather than the patient’s residential address.

### 2.3. Statistical Analysis

Two analytic units were used in this study. Unique claims-defined episodes were used to describe monthly patterns. Each episode was assigned to the month of its index date and counted once in the diagnostic category first recorded chronologically during the episode.

Diagnostic category records were used for all other category-specific analyses. An individual patient could contribute multiple episodes, and an episode containing qualifying codes from more than one diagnostic category contributed once to each applicable category. Consequently, the number of diagnostic category records could exceed the number of unique claims-defined episodes. Diagnostic category records were summarized using frequencies and percentages according to diagnostic category, sex, age group, insurance type, selected underlying disease, healthcare institution type, and region. Percentages were calculated using the total number of records within the corresponding diagnostic category column as the denominator.

For regional analyses, crude annual healthcare-institution-location-based diagnostic category record rates per 100,000 regional residents were calculated by dividing the number of diagnostic category records associated with healthcare institutions in each administrative region by the corresponding 2023 regional resident population reported by Statistics Korea and multiplying by 100,000. These measures were descriptive healthcare-utilization rates based on healthcare institution location and were not interpreted as patient residential incidence rates. The rates were not adjusted for regional age structure, healthcare accessibility, patient mobility, or other population characteristics. Regional distributions were visualized using choropleth maps based on administrative districts.

As a sensitivity analysis, selected descriptive summaries were repeated after excluding the unspecified diagnostic category defined by KCD-8 code A09.0. This analysis was conducted to describe the distributions of records assigned to more specific diagnostic categories and was not intended to validate episodes assigned to the unspecified category.

No hypothesis testing, regression modeling, or causal inference was performed because the study objective was descriptive. All analyses were conducted using SAS software version 9.4 (SAS Institute Inc., Cary, NC, USA).

## 3. Results

A total of 6,829,577 unique claims-defined foodborne illness-related episodes were identified in South Korea during 2023. Because an episode could contain qualifying codes from more than one diagnostic category, these episodes generated 7,754,215 diagnostic category records. Of these records, 6,147,343 (79.3%) were classified as unspecified, 935,086 (12.1%) as bacterial, 668,057 (8.6%) as viral, and 3729 (<0.1%) as protozoal/other-cause. Female and male records accounted for 3,996,581 (51.5%) and 3,757,634 (48.5%), respectively.

The distribution of diagnostic category records differed across age groups. Children aged <2 years accounted for 1,236,778 records (15.9%), those aged 2–5 years for 1,645,055 (21.2%), those aged 6–11 years for 984,400 (12.7%), and those aged 12–18 years for 536,860 (6.9%). Adults aged 19–64 years accounted for 2,501,300 records (32.3%), and adults aged ≥65 years accounted for 849,822 (11.0%). Viral and unspecified records were more frequently observed in younger age groups, whereas most bacterial records occurred among adults aged 19–64 years. Because age-specific population denominators were not used, these distributions should not be interpreted as age-specific incidence or risk (Table 1).

Most diagnostic category records were associated with clinic visits (6,227,416, 80.3%), followed by hospitals (897,464, 11.6%), general hospitals (507,041, 6.5%), tertiary hospitals (72,508, 0.9%), and other institutions (49,786, 0.6%). The unspecified category accounted for the largest number of records in all healthcare institution types. Among records assigned to more specific diagnostic categories, bacterial records outnumbered viral records in clinics, general hospitals, and tertiary hospitals, whereas viral records outnumbered bacterial records in hospitals (Table 1).

Records occurring among patients with selected underlying diseases represented a relatively small subset of all diagnostic category records. Diabetes mellitus was the most frequently identified selected underlying condition, followed by malignant neoplasms and organ transplantation. No qualifying claims-defined foodborne illness-related episodes were identified among patients with HIV/AIDS. Records among patients with diabetes mellitus or malignant neoplasms were concentrated in older age groups, whereas records among organ transplant recipients occurred primarily among adults aged 19–64 years. The distribution by healthcare institution type also differed across the selected conditions. Records among patients with diabetes mellitus were most frequently associated with clinics, whereas those among patients with malignant neoplasms or organ transplantation were more frequently associated with general or tertiary hospitals. These findings describe the healthcare settings in which the records occurred and should not be interpreted as measures of clinical severity. Detailed distributions by age group, diagnostic category, healthcare institution type, and episode numbers are presented in Supplementary Table S2.

**Figure 1:**
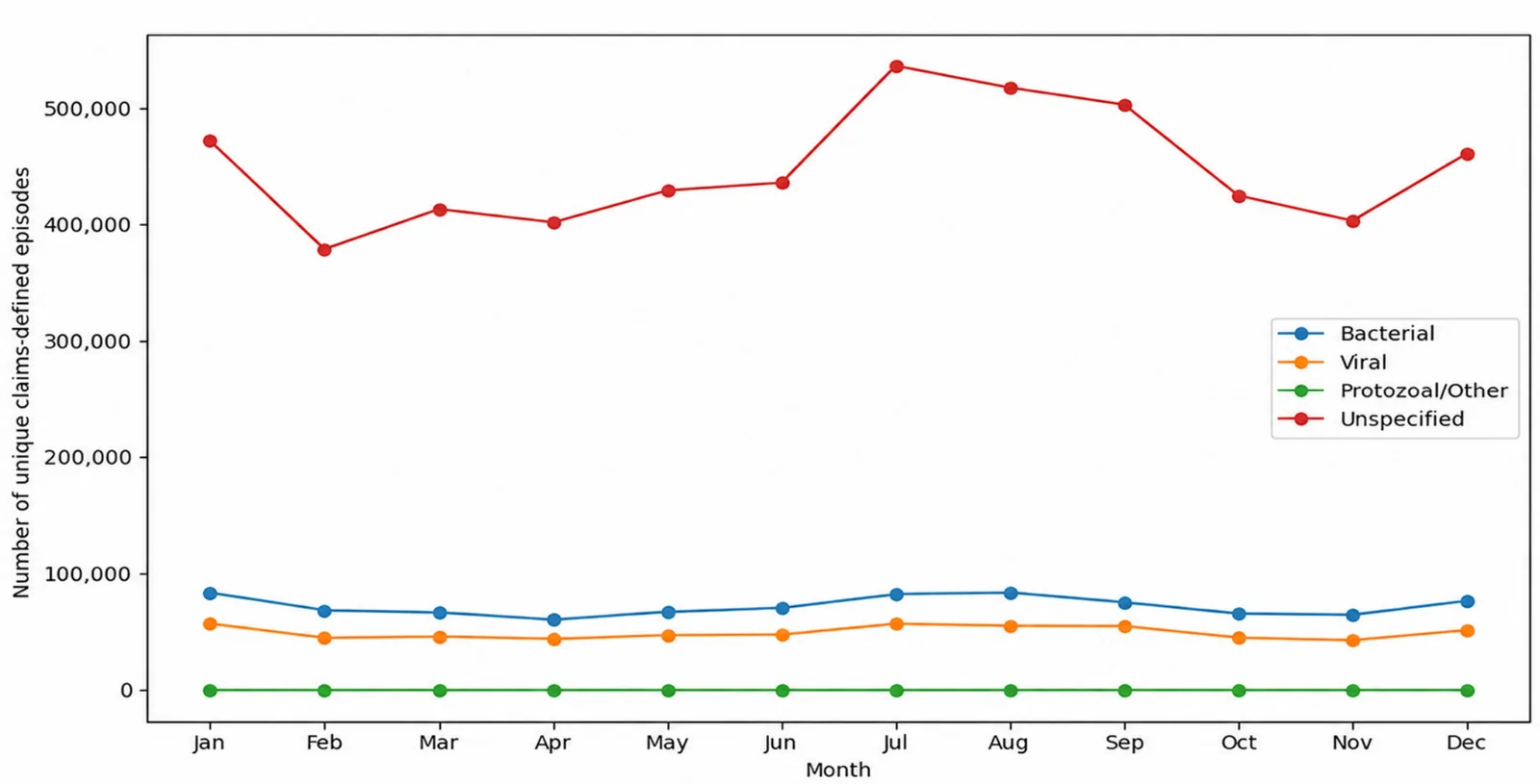
Monthly distribution of unique claims-defined foodborne illness-related episodes by first-recorded diagnostic category in South Korea, 2023.

Monthly variation was observed in the counts of unique claims-defined foodborne illness-related episodes during 2023. The lowest monthly count was observed in February (492,348), and the highest count was observed in July (674,768). Counts were also relatively high in January (611,497), August (655,905), and September (632,692). Monthly episode counts according to the diagnostic category first recorded during each episode are presented in Supplementary Table S3 and Figure 1.

**Figure 2:**
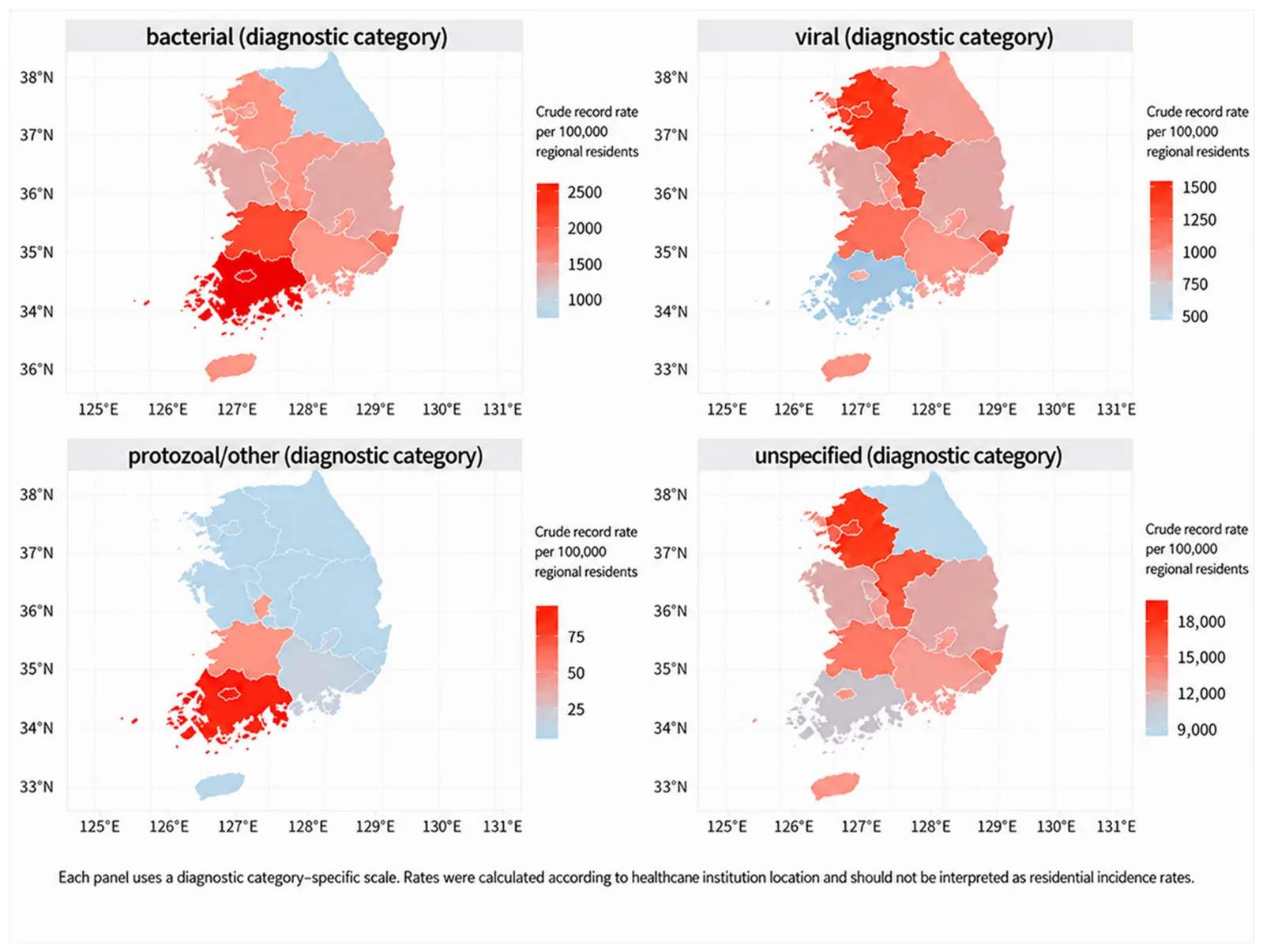
Crude diagnostic category record rate per 100,000 regional residents. Rates were calculated according to healthcare institution location using category-specific scales and should not be interpreted as residential incidence rates.

Crude annual diagnostic category record rates based on healthcare institution location varied across administrative regions and diagnostic categories (Figure 2). Bacterial records showed relatively high rates in Jeollanam-do, Jeollabuk-do, and Ulsan, whereas viral records showed relatively high rates in Gwangju and Gyeongsangnam-do. Protozoal/other-cause records were uncommon, with the highest rate observed in Jeollanam-do. The unspecified category showed the highest rate in Sejong-si. These rates were based on healthcare institution location rather than patient residence and were not adjusted for regional age structure, healthcare accessibility, or patient mobility.

After excluding the unspecified diagnostic category, the distributions by sex and healthcare institution type were generally comparable with those observed in the main analysis. The results of the sensitivity analysis are presented in Supplementary Table S4.

## 4. Discussion

This nationwide claims-based descriptive study characterized administrative diagnostic coding and healthcare-utilization patterns for foodborne illness-related conditions documented in the 2023 HIRA database. Monthly variation was assessed using unique episodes, with each episode counted once according to the diagnostic category first recorded during the episode, whereas distributions across age groups, healthcare institution types, selected underlying diseases, and regions were described using diagnostic category records. Most diagnostic category records were assigned to the unspecified category and were documented in clinics, while records occurring among patients with the selected underlying diseases represented a relatively small proportion of all records. These findings describe the volume and distribution of administrative diagnostic records and their distribution across healthcare settings and should not be interpreted as evidence of confirmed disease incidence, etiology, individual-level risk, clinical severity, or healthcare system burden.

The unspecified diagnostic category accounted for approximately four-fifths of all diagnostic category records, indicating that routine claims coding was predominantly nonspecific rather than assigned to one of the more specific bacterial, viral, or protozoal/other categories included in this study. Diagnostic codes in administrative claims are primarily generated for clinical documentation and reimbursement and may not correspond to etiologically confirmed diagnoses [8,9,10,15,16]. Previous research on foodborne illness has also emphasized that etiologic confirmation is limited by multiple steps, including healthcare seeking, specimen submission, laboratory testing, and pathogen identification [17]. However, the present claims data did not allow us to determine whether unspecified codes were used because testing was not performed, results were unavailable at the time of coding, a specific cause could not be identified, or a nonspecific code was considered sufficient for clinical documentation. The predominance of unspecified records should therefore be interpreted primarily as a feature of diagnostic recording in routine healthcare practice rather than as evidence regarding the etiologic composition of foodborne illness in the population.

The distribution of diagnostic category records differed across age groups. Viral and unspecified records were more frequently observed in younger age groups, whereas bacterial records were concentrated among adults aged 19–64 years. Similar age-related differences have been reported in surveillance-based studies of sporadic foodborne disease, although the observed distributions vary by pathogen, study population, surveillance system, and case definition [18]. Direct comparison with such studies is limited because the present analysis used claims-defined records rather than laboratory-confirmed or actively monitored cases. In addition, age-specific population denominators were not applied. The findings therefore indicate which age groups contributed larger proportions of recorded healthcare encounters within each diagnostic category but do not demonstrate differences in age-specific incidence or susceptibility. Healthcare-seeking behavior, diagnostic practices, testing patterns, and coding practices may have contributed to the observed distributions, but these factors could not be evaluated using the available data [15,16].

More than 80% of all diagnostic category records were associated with clinic visits. This finding indicates that most qualifying diagnostic records in the claims database were generated in outpatient clinical settings rather than in general or tertiary hospitals. It does not, however, establish that most episodes were clinically mild. The institution-level distribution may reflect healthcare access and referral patterns in addition to clinical condition [8,9]. However, institution type alone does not identify medical specialty or specialty-specific healthcare-seeking pathways. Furthermore, the dataset did not contain standardized measures of symptom intensity, dehydration, laboratory abnormalities, complications, or clinical outcomes. Because eligible diagnostic codes could appear in either the primary or secondary diagnosis position, the institution-level distribution reflects where qualifying records were documented and reimbursed rather than a validated classification of disease severity.

Records among patients with diabetes mellitus, malignant neoplasms, or organ transplantation accounted for a relatively small subset of all diagnostic category records. Their distributions differed by age group and healthcare institution type. Records among patients with diabetes were most frequently associated with clinics, whereas records among patients with malignant neoplasms or organ transplantation were more frequently documented in general or tertiary hospitals. These findings describe the demographic and healthcare-setting characteristics of patients with qualifying diagnostic records. Because the study lacked population denominators for the respective underlying-disease groups and an appropriate comparison population, and did not adjust for background disease prevalence or other potential confounders, it cannot determine whether patients with these conditions had a higher likelihood of foodborne illness-related healthcare encounters. The absence of validated clinical outcomes also precluded assessment of clinical severity. The results should therefore not be interpreted as evidence of a causal relationship, increased susceptibility, or adverse prognosis.

Monthly counts of unique claims-defined episodes varied during 2023, with the highest count observed in July and relatively high counts also observed in January, August, and September. Previous Korean outbreak surveillance studies have reported temporal variation in waterborne and foodborne disease outbreaks, including warmer-month concentrations of several bacterial outbreaks and winter activity of norovirus [5,6,19,20]. Studies based on sporadic foodborne disease monitoring have likewise shown that temporal distributions can differ substantially across pathogens [18]. These studies provide context for the monthly variation observed in the present analysis, but they are not directly comparable because they were based on outbreak surveillance or pathogen-specific monitoring. The relatively high counts observed in both summer and winter may reflect the inclusion of heterogeneous diagnostic categories with different temporal distributions. Because the present study included only one calendar year and lacked microbiological results and sufficient diagnostic specificity, the observed monthly differences could not be attributed to particular pathogens and may reflect year-specific fluctuations, healthcare-seeking behavior, diagnostic practices, and post-pandemic changes in mobility, dining behavior, international travel, and healthcare utilization. The findings should therefore be interpreted as within-year monthly variation rather than established seasonality. Multi-year claims data linked, where possible, with laboratory or surveillance information would be required to assess the reproducibility and possible determinants of these temporal patterns.

The relatively high counts observed in both summer and winter may reflect the inclusion of heterogeneous diagnostic categories with different temporal distributions. Nevertheless, the present study did not contain microbiological results or sufficient diagnostic specificity to attribute the monthly counts to particular pathogens. Moreover, data from one calendar year are insufficient to determine whether the observed monthly distribution represents a stable and recurring seasonal pattern. The findings should therefore be described as within-year monthly variation rather than established seasonality. Furthermore, because 2023 followed substantial relaxation of COVID-19-related restrictions, the observed monthly distribution may partly reflect year-specific post-pandemic changes in mobility, dining behavior, international travel, healthcare-seeking behavior, and healthcare utilization. Multi-year claims data linked, where possible, with laboratory or surveillance information would be needed to assess the reproducibility and possible determinants of these temporal patterns.

Crude annual diagnostic category record rates varied across administrative regions. However, these rates were calculated according to the location of the healthcare institution at which each record was generated rather than the patient’s residential location. They therefore describe the geographic distribution of healthcare-recorded diagnostic records and related healthcare utilization, not the geographic distribution of disease occurrence or regional incidence among residents. Patients may obtain care outside their residential region, and the extent of such mobility may differ according to healthcare accessibility and the availability and type of healthcare institutions. Regional differences may also be influenced by healthcare institution density, diagnostic testing, and coding practices. Consequently, the observed regional differences should not be interpreted as differences in the underlying regional risk of foodborne illness.

After exclusion of the unspecified diagnostic category, the distributions by sex and healthcare institution type were generally comparable with those observed in the primary analysis. This sensitivity analysis provides an additional description of records assigned to more specific diagnostic categories. However, exclusion of unspecified records does not establish that the remaining bacterial, viral, or protozoal/other records were microbiologically confirmed. Studies evaluating Korean health insurance claims have shown that diagnostic codes alone may be subject to misclassification and that algorithms incorporating additional clinical or reimbursement information can improve case identification for some conditions [15]. The sensitivity analysis should therefore be regarded as a complementary descriptive summary rather than validation of the etiologic classification.

Several limitations should be considered. First, claims diagnoses were not linked to microbiological test results, food-exposure histories, epidemiological investigations, or validated clinical outcomes. Therefore, the records could not be confirmed as foodborne illnesses or attributed to specific foods, pathogens, transmission routes, or outbreak events. Furthermore, the study did not analyze costs, length of stay, emergency department use, number of encounters per episode, diagnostic procedures, treatment intensity, complications, or clinical outcomes. Accordingly, counts of claims-defined episodes and diagnostic category records should not be interpreted as measures of epidemiological or healthcare system burden; such assessments would require linked clinical, microbiological, epidemiological, and economic data. Second, the identification of episodes and diagnostic categories was based on operational claims-based definitions. For analyses based on diagnostic category records, the diagnostic categories were not mutually exclusive, and a single episode could contribute to more than one category. Because the 30-day interval was an operational claims-based definition rather than a clinically validated threshold for foodborne illness, it may have combined distinct acute illnesses occurring within 30 days or separated prolonged episodes. Accordingly, diagnostic category record counts should not be interpreted as numbers of unique episodes or affected individuals. Third, both primary and secondary diagnosis positions were used to identify eligible episodes. Consequently, some qualifying codes recorded as secondary diagnoses may have represented comorbid, provisional, or incidental conditions rather than the principal reason for the healthcare encounter. Because a primary-diagnosis-only sensitivity analysis was not performed, the extent to which inclusion of secondary diagnoses affected the number and distribution of claims-defined episodes could not be determined [15,16]. Fourth, population-based denominators were not available for most demographic and clinical subgroups, and regional rates were based on healthcare institution location rather than patient residence. These rates were not adjusted for population structure, healthcare accessibility, healthcare institution availability and density, diagnostic practices, or patient mobility and therefore should not be interpreted as geographic differences in disease occurrence or underlying regional risk. Finally, the study covered only one calendar year and therefore could not assess annual or long-term trends or determine whether the observed monthly variation represented a recurring seasonal pattern. Because 2023 followed substantial relaxation of COVID-19-related restrictions, the observed patterns may also partly reflect year-specific post-pandemic changes and may not represent typical long-term patterns.

Nevertheless, this study has several important strengths. It used nationwide claims data covering healthcare encounters across a broad range of patients and healthcare institutions in South Korea, thereby providing a population-wide description of diagnostic recording and healthcare utilization in routine clinical practice. Such data offer information that is complementary to outbreak-based and laboratory-based surveillance, particularly by capturing medically attended records that may not be linked to recognized outbreaks [8,9,10]. The episode-based approach reduced repeated counting of closely spaced encounters for the same patient, and the study explicitly distinguished diagnostic category records from unique episodes and individual patients. This transparent definition and reporting of the unit of analysis is particularly important in studies using routinely collected healthcare data [21].

## 5. Conclusions

This nationwide claims-based study described administrative diagnostic coding and healthcare-utilization patterns for foodborne illness-related conditions across demographic groups, healthcare settings, calendar months, and regions in South Korea during 2023, a post-pandemic transition period. Most diagnostic category records were unspecified and were documented in clinics, indicating limited etiologic specificity in routine diagnostic coding and the predominance of outpatient documentation. These findings provide a complementary perspective on medically attended foodborne illness-related conditions that may not be captured through outbreak-based or laboratory-based surveillance alone. From a public health perspective, greater etiologic specificity and linkage of claims data with laboratory and public health surveillance information may improve the interpretation and utility of routinely collected healthcare data. Nevertheless, these findings should not be interpreted as estimates of confirmed disease incidence or etiology or as evidence of recurring seasonal patterns or long-term epidemiological trends.

## Figures and Tables

**Table 1:** Demographic and healthcare utilization characteristics of diagnostic category records generated from claims-defined foodborne illness-related episodes.

Characteristic			Bacterial Records, *n* = 935,086 (%)	Viral Records, *n* = 668,057 (%)	Protozoal/Other Records, *n* = 3729 (%)	Unspecified Records, *n* = 6,147,343 (%)	All Diagnostic Category Records, *n* = 7,754,215 (%)
Sex	Female		506,440 (54.2)	343,303 (51.4)	1890 (50.7)	3,144,948 (51.2)	3,996,581 (51.5)
	Male		428,646 (45.8)	324,754 (48.6)	1839 (49.3)	3,002,395 (48.8)	3,757,634 (48.5)
Age group	Pediatric	<2	18,037 (1.9)	108,886 (16.3)	26 (0.7)	1,109,829 (18.1)	1,236,778 (15.9)
		2–5	25,777 (2.8)	145,708 (21.8)	61 (1.6)	1,473,509 (24.0)	1,645,055 (21.2)
		6–11	35,385 (3.8)	114,006 (17.1)	160 (4.3)	834,849 (13.6)	984,400 (12.7)
		12–18	65,172 (7.0)	60,963 (9.1)	201 (5.4)	410,524 (6.7)	536,860 (6.9)
	Adult	19–64	563,098 (60.2)	183,543 (27.5)	2269 (60.8)	1,752,390 (28.5)	2,501,300 (32.3)
	Elderly	≥65	227,617 (24.3)	54,951 (8.2)	1012 (27.1)	566,242 (9.2)	849,822 (11.0)
Insurance type	National Health Insurance		897,926 (96.0)	653,428 (97.8)	3586 (96.2)	6,005,633 (97.7)	7,560,573 (97.5)
	Medical Aid		37,160 (4.0)	14,629 (2.2)	143 (3.8)	141,710 (2.3)	193,642 (2.5)
Healthcare institution type	Clinic		814,796 (87.1)	579,740 (86.8)	2621 (70.3)	4,830,259 (78.6)	6,227,416 (80.3)
	Hospital		38,795 (4.1)	57,154 (8.6)	215 (5.8)	801,300 (13.0)	897,464 (11.6)
	General hospital		56,486 (6.0)	23,826 (3.6)	570 (15.3)	426,159 (6.9)	507,041 (6.5)
	Tertiary hospital		20,780 (2.2)	5205 (0.8)	300 (8.0)	46,223 (0.8)	72,508 (0.9)
	Other institutions		4229 (0.5)	2132 (0.3)	23 (0.6)	43,402 (0.7)	49,786 (0.6)

Note: A total of 6,829,577 unique claims-defined episodes generated 7,754,215 diagnostic category records because an episode containing qualifying codes from more than one diagnostic category was included once in each applicable category. Percentages were calculated within each diagnostic category column. The “All diagnostic category records” column represents the sum of diagnostic category assignments and should not be interpreted as the number of unique episodes or individuals.

## Data Availability

The data that support the findings of this study are not publicly available because of privacy and ethical restrictions imposed by the Health Insurance Review and Assessment Service (HIRA).

## Supplementary Materials

The following supporting information can be downloaded at: https://www.mdpi.com/article/10.3390/healthcare14162466/s1, Table S1: Diagnostic Code Definitions for Claims-Based Diagnostic Categories Used in This Study; Table S2: Age and Healthcare Institution Distributions of Diagnostic Category Records among Selected Underlying-Disease Cohorts; Table S3: Monthly Distribution of Unique Claims-Defined Foodborne Illness-Related Episodes According to the Diagnostic Category First Recorded during Each Episode; Table S4: Demographic and Healthcare Utilization Characteristics of Foodborne Illness-Related Diagnostic Category Records after Excluding the Unspecified Diagnostic Category (A09.0).

## Abbreviations

The following abbreviations are used in this manuscript:

HIRA	Health insurance review and assessment service
KCD	Korean standard classification of diseases and causes of death
