# Supplementary material for: Foodborne Illness-Related Diagnostic Coding and Healthcare-Utilization Patterns in South Korea, 2023: A Nationwide Claims-Based Descriptive Study
Source: Healthcare (Basel). 2026 Aug 10;14(16):2466. doi: 10.3390/healthcare14162466 (PMC13512080; doi:10.3390/healthcare14162466)
Supplement: Supplementary file 1 [file healthcare-14-02466-s001.zip › healthcare-4443587-supplementary.pdf]

Table S1. Diagnostic Code Definitions for Claims-Based Diagnostic Categories Used in This Study.

Classification	Diagnostic codes	Specificity for foodborne transmission
Bacterial diagnostic category	Salmonella enteritis (A02.0), Enteropathogenic Escherichia coli infection (A04.0), Enterotoxigenic Escherichia coli infection (A04.1), Enteroinvasive Escherichia coli infection (A04.2), Enterohemorrhagic Escherichia coli infection (A04.3), Other intestinal Escherichia coli infection (A04.4), Enterocolitis due to Clostridioides difficile (A04.7), Staphylococcal foodborne illness (A05.0), Botulism (A05.1), Foodborne *Clostridium perfringens* intoxication (A05.2), Foodborne *Vibrio parahaemolyticus* intoxication (A05.3), Foodborne *Bacillus cereus* intoxication (A05.4), Other specified bacterial foodborne intoxications (A05.8), Bacterial foodborne intoxication, unspecified (A05.9)	Relatively specific
Viral diagnostic category	Rotaviral enteritis (A08.0), Norovirus infection (A08.1), Adenoviral enteritis (A08.2), Other viral enteritis (A08.3)	Relatively specific
Protozoal/other-cause diagnostic category	Balantidiasis (A07.0), Giardiasis (A07.1), Cryptosporidiosis (A07.2), Isosporiasis (A07.3), Toxicity from other harmful substances ingested as food (T62)	Relatively specific

Table S2. Age and healthcare institution distributions of diagnostic-category records among selected underlying-disease cohorts.

characteristic				Bacterial	Viral	Protozoal/other	Unspecified	total
DM	Age	Pediatric	under 2	0 (0.0)	6 (0.4)	0 (0.0)	14 (0.1)	20 (0.1)
			2-5	1 (0.0)	5 (0.3)	0 (0.0)	25 (0.1)	31 (0.1)
			6-11	6 (0.1)	6 (0.4)	0 (0.0)	40 (0.2)	52 (0.2)
			12-18	38 (0.5)	27 (1.8)	0 (0.0)	141 (0.7)	206 (0.7)
			total	45 (0.6)	44 (3.0)	0 (0.0)	220 (1.0)	309 (1.0)
		Adult	19-64	2,951 (37.6)	530 (36.2)	6 (26.1)	8,718 (41.6)	12,205 (40.3)
		Elderly	≥65	4,845 (61.8)	891 (60.8)	17 (73.9)	12,022 (57.4)	17,775 (58.7)
	Healthcare institution type	Clinic		6,042 (77.1)	1,133 (77.3)	11 (47.8)	13,724 (65.5)	20,910 (69.0)
		Hospital		490 (6.2)	53 (3.6)	0 (0.0)	2,445 (11.7)	2,988 (9.9)
		General hospital		1,045 (13.3)	236 (16.1)	12 (52.2)	4,417 (21.1)	5,710 (18.9)
		Tertiary hospital		201 (2.6)	40 (2.7)	0 (0.0)	240 (1.1)	481 (1.6)
		Other institutions		63 (0.8)	3 (0.2)	0 (0.0)	134 (0.6)	200 (0.7)
		Total		7841 (25.9)	1,465 (4.8)	23 (0.1)	20,960 (69.2)	30,289 (100.0)
TPL	Age	Pediatric	under 2	0 (0.0)	5 (12.8)	0 (0.0)	5 (1.3)	10 (1.6)
			2-5	0 (0.0)	2 (5.1)	0 (0.0)	2 (0.5)	4 (0.6)
			6-11	1 (0.5)	1 (2.6)	0 (0.0)	6 (1.6)	8 (1.3)
			12-18	3 (1.4)	0 (0.0)	0 (0.0)	1 (0.3)	4 (0.6)
			total	4 (1.8)	8 (20.5)	0 (0.0)	14 (3.7)	26 (4.1)
		Adult	19-64	147 (67.1)	29 (74.4)	0 (0.0)	251 (67.1)	427 (67.6)
		Elderly	≥65	68 (31.1)	2 (5.1)	0 (0.0)	109 (29.1)	179 (28.3)
	Healthcare institution type	Clinic		7 (3.2)	3 (7.7)	0 (0.0)	24 (6.4)	34 (5.4)
		Hospital		0 (0.0)	1 (2.6)	0 (0.0)	13 (3.5)	14 (2.2)
		General hospital		29 (13.2)	9 (23.1)	0 (0.0)	73 (19.5)	111 (17.6)
		Tertiary hospital		183 (83.6)	26 (66.7)	0 (0.0)	264 (70.6)	473 (74.8)
		Other institutions		0 (0.0)	0 (0.0)	0 (0.0)	0 (0.0)	0 (0.0)
		Total		219 (34.7)	39 (6.2)	0 (0.0)	374 (59.2)	632 (100.0)

characteristic				Bacterial	Viral	Protozoal/other	Unspecified	total
Cancer	Age	Pediatric	under 2	2 (0.0)	6 (1.4)	0 (0.0)	21 (0.2)	29 (0.2)
			2-5	12 (0.2)	19 (4.4)	0 (0.0)	35 (0.4)	66 (0.4)
			6-11	32 (0.5)	5 (1.2)	0 (0.0)	27 (0.3)	64 (0.4)
			12-18	15 (0.2)	3 (0.7)	0 (0.0)	25 (0.3)	43 (0.3)
			total	61 (1.0)	33 (7.7)	0 (0.0)	108 (1.1)	202 (1.2)
		Adult	19-64	2,463 (39.7)	160 (37.4)	4 (30.8)	3,929 (39.4)	6,556 (39.5)
		Elderly	≥65	3,682 (59.3)	235 (54.9)	9 (69.2)	5,931 (59.5)	9,857 (59.3)
	Healthcare institution type	Clinic		1,317 (21.2)	192 (44.9)	1 (7.7)	3,260 (32.7)	4,770 (28.7)
		Hospital		176 (2.8)	18 (4.2)	2 (15.4)	1,197 (12.0)	1,393 (8.4)
		General hospital		1,540 (24.8)	87 (20.3)	1 (7.7)	3,321 (33.3)	4,949 (29.8)
		Tertiary hospital		3,051 (49.2)	118 (27.6)	9 (69.2)	2,016 (20.2)	5,194 (31.3)
		Other institutions		122 (2.0)	13 (3.0)	0 (0.0)	174 (1.7)	309 (1.9)
	Total			6,206 (37.4)	428 (2.6)	13 (0.1)	9,968 (60.0)	16,615 (100.0)

DM, diabetes mellitus; TPL, transplantation. No qualifying claims-defined foodborne illness-related episodes were identified among patients with HIV/AIDS; therefore, this cohort is not displayed in the table.

Table S3. Monthly distribution of unique claims-defined foodborne illness-related episodes according to the diagnostic category first recorded during each episode.

Month	Bacterial	Viral	protozoal/other	Unspecified	Total
January	82,936 (13.6)	56,770 (9.3)	302 (0.0)	471,489 (77.1)	611,497 (100.0)
February	68,267 (13.9)	44,977 (9.1)	271 (0.1)	378,833 (76.9)	492,348 (100.0)
March	66,363 (12.6)	45,862 (8.7)	386 (0.1)	412,827 (78.6)	525,438 (100.0)
April	60,160 (11.9)	44,176 (8.7)	335 (0.1)	401,667 (79.3)	506,338 (100.0)
May	66,877 (12.3)	47,172 (8.7)	292 (0.1)	428,920 (79.0)	543,261 (100.0)
June	69,982 (12.7)	47,576 (8.6)	245 (0.0)	435,396 (78.7)	553,199 (100.0)
July	81,751 (12.1)	56,691 (8.4)	308 (0.0)	536,018 (79.4)	674,768 (100.0)
August	82,959 (12.6)	55,204 (8.4)	289 (0.0)	517,453 (78.9)	655,905 (100.0)
September	74,960 (11.8)	54,723 (8.6)	313 (0.0)	502,696 (79.5)	632,692 (100.0)
October	65,366 (12.2)	45,071 (8.4)	285 (0.1)	424,429 (79.3)	535,151 (100.0)
November	64,480 (12.6)	43,033 (8.4)	215 (0.0)	403,021 (78.9)	510,749 (100.0)
December	76,136 (12.9)	51,464 (8.7)	280 (0.0)	460,351 (78.3)	588,231 (100.0)
Total	860,237 (12.6)	592,719 (8.7)	3,521 (0.1)	5,373,100 (78.7)	6,829,577 (100.0)

Note: *Each claims-defined episode was assigned to the month of its index date and counted once in the diagnostic category first recorded chronologically during the episode. Percentages were calculated using the total number of unique episodes in each month as the denominator.*

Table S4. Demographic and healthcare utilization characteristics of foodborne illness-related diagnostic category records after excluding the unspecified diagnostic category (A09.0)

Characteristic			Bacterial n=935,086 (%)	Viral n=668,057 (%)	Protozoal/other n=3,729 (%)	Total n=1,606,872 (%)
Sex	Female		506,440 (54.2)	343,303 (51.4)	1,890 (50.7)	851,633 (53.0)
	Male		428,646 (45.8)	324,754 (48.6)	1,839 (49.3)	755,239 (47.0)
Age group	Pediatric	<2	18,037 (1.9)	108,886 (16.3)	26 (0.7)	126,949 (7.9)
		2–5	25,777 (2.8)	145,708 (21.8)	61 (1.6)	171,546 (10.7)
		6–11	35,385 (3.8)	114,006 (17.1)	160 (4.3)	149,551 (9.3)
		12–18	65,172 (7.0)	60,963 (9.1)	201 (5.4)	126,336 (7.9)
	Adult	19–64	563,098 (60.2)	183,543 (27.5)	2,269 (60.8)	748,910 (46.6)
	Elderly	≥65	227,617 (24.3)	54,951 (8.2)	1,012 (27.1)	283,580 (17.6)
Insurance type	Health insurance		897,926 (96.0)	653,428 (97.8)	3,586 (96.2)	1,554,940 (96.8)
	Medical Aid		37,160 (4.0)	14,629 (2.2)	143 (3.8)	51,932 (3.2)
Hospital level	Clinic		814,796 (87.1)	579,740 (86.8)	2,621 (70.3)	1,397,157 (86.9)
	Hospital		38,795 (4.1)	57,154 (8.6)	215 (5.8)	96,164 (6.0)
	General hospital		56,486 (6.0)	23,826 (3.6)	570 (15.3)	80,882 (5.0)
	Tertiary hospital		20,780 (2.2)	5,205 (0.8)	300 (8.0)	26,285 (1.6)
	Other institutions		4,229 (0.5)	2,132 (0.3)	23 (0.6)	6,384 (0.4)
